# New PCR systems to confirm real-time PCR detection of *Mycobacterium avium *subsp. *paratuberculosis*

**DOI:** 10.1186/1471-2180-6-87

**Published:** 2006-10-04

**Authors:** David Herthnek, Göran Bölske

**Affiliations:** 1 National Veterinary Institute (SVA), Department of Bacteriology, SE-751 89 Uppsala, Sweden

## Abstract

**Background:**

Johne's disease, a serious chronic form of enteritis in ruminants, is caused by *Mycobacterium avium *subsp. *paratuberculosis *(MAP). As the organism is very slow-growing and fastidious, several PCR-based methods for detection have been developed, based mainly on the MAP-specific gene IS900. However, because this gene is similar to genes in other mycobacteria, there is a need for sensitive and reliable methods to confirm the presence of MAP. As described here, two new real-time PCR systems on the IS900 gene and one on the F57 gene were developed and carefully validated on 267 strains and 56 positive clinical faecal samples.

**Results:**

Our confirmatory PCR systems on IS900 were found sensitive and specific, only yielding weak false positive reactions in one strain for each system. The PCR system on F57 did not elicit any false positives and was only slightly less sensitive than our primary IS900-system. DNA from both naturally infected and spiked faeces that tested positive with our primary system could be confirmed with all new systems, except one low-level infected sample that tested negative with the F57 system.

**Conclusion:**

We recommend using the newly constructed DH3 PCR system on the F57 gene as the primary confirmatory test for PCR positives, but should it fail due to its lower sensitivity, the DH1 and DH2 PCR systems should be used.

## Background

Paratuberculosis (Johne's disease) is caused by *Mycobacterium avium *subsp. *paratuberculosis *(MAP). It is a ubiquitous chronic enteric wasting disease of ruminants, though in Sweden the disease is rare or absent, thanks to successful control measures in the past. It is regarded as an exotic disease and falls under the Swedish Epizootic Act, which means that in an event of an outbreak, measures must be taken promptly to combat the disease and to trace the origin of the outbreak [1,2]. When a positive case is identified, consequences for the farmer are grave and it is usually deemed necessary to slaughter the whole herd.

In the Swedish Paratuberculosis Control Program and in most of the Swedish surveillances undertaken to monitor freedom from paratuberculosis, detection is based on culture. During the culture procedure, suspected colonies are picked and identified as MAP by PCR. Other characteristics of the colony isolate act to confirm the identification made with PCR, namely acid-fast staining (acid-fast bacilli), growth characteristics (small, slow-growing) and dependence on mycobactin. The PCR methods generally used to identify and detect MAP are based on IS900, an insertion sequence considered specific for MAP [3-6]. IS900 is a 1,451 bp segment that lacks inverted terminal repeats and does not generate direct repeats in target DNA [7]. It belongs to the same family of insertion sequences as IS901, IS902, and IS1110, described in *M. avium *subsp. *avium*, *M. avium *subsp. *silvaticum*, and *M. avium *subsp. *avium*, respectively [8-10].

PCR based on IS900 has been used for direct detection of MAP, without primary culture, from milk, faecal specimens, semen, and human intestinal tissue [11-16]. Apart from being the method of choice when speed is a priority, direct PCR is also preferred when MAP is difficult or impossible to cultivate [17].

However, as IS900-like genes have been found in other unrelated *Mycobacterium *species, it is evident that the PCR systems used for IS900 are not completely specific for MAP [18-20]. In a recent investigation, a nested PCR on IS900 was shown to elicit false positive reactions from several mycobacterial strains [21]. It is therefore desirable to use alternative PCR systems to confirm a positive IS900 PCR for MAP. This is useful when PCR identification cannot be confirmed by conventional culture-based methods, as is the case with direct PCR and PCR identification of growth in liquid cultures [22,23].

In the present paper, two new real-time PCR systems that target other parts of IS900 than does our standard (primary) PCR [16], and one real-time PCR targeting the F57 gene, specific for MAP [24], were developed and evaluated as confirming tests on strains and clinical samples, previously found positive with our standard IS900 PCR.

## Results

### Specificity

All MAP strains and isolates (Table 1) proved positive with the primary PCR system and were confirmed by all three confirmatory systems. Listed in Table 2 are a variety of other mycobacteria, as well as a few other bacterial strains, that were tested with all four real-time PCR systems. With the primary PCR system, strain 2333 proved positive, as previously reported [20]. An isolate of *Mycobacterium avium *subsp. *avium *from a Swedish horse and a *Mycobacterium kubicae*-related isolate from cat gave weak positive reactions (i.e. high CT values) with the IS900 systems DH1 and DH2, respectively. All the other strains tested negative. The F57 system DH3 gave no false positive reactions.

### Direct PCR on faecal samples

The procedure of first detecting MAP (or noting PCR inhibition) with the primary system, then confirming positives with the three other new systems, worked well with direct PCR on clinical samples. When inhibitors were still present, which only was the case with samples from the External Quality Assessment (indicated in 14 of 52 samples), a dilution of the DNA or a new preparation of the original sample would usually solve the problem and inhibition only remained in three of the samples. Our method could detect MAP in 27 of the 28 valid positive samples in the 52 samples from the External Quality Assessment. When a sample produced a positive signal with the primary system, the same result was obtained with the confirmatory systems. However, as shown in Table 3, for 12 of 56 positive samples, re-runs with additional replicates had to be performed on F57 in order to pick up the dispersed DNA, and for one culture-negative clinical sample with weak IS900 PCR signals, DH3 failed to confirm the presence of MAP.

MAP could be detected in the spiked faeces using the primary system at a spiking level of 10^4 ^organisms/g and readily confirmed at the same concentration by all three confirmatory systems without the need for re-runs.

### Sensitivity on pure DNA

The IS900 systems MP, DH1, DH2 and the F57 system DH3 yielded positive signals from DNA suspensions with the concentrations 0.1, 0.1, 0.3 and 1 MAP genomes/μl respectively.

## Discussion

Real-time PCR is a sensitive method for detection that eliminates the need to open the tubes when analysing the product, a stage in other PCR techniques that often constitutes a risk of cross-contamination. In particular, the sensitive method nested PCR involves a great risk of contaminating other samples when the product from its first reaction is being transferred to the second. Real-time PCR minimises the risk of false positive results due to amplicon contamination.

Another advantage of real-time PCR is that the use of probes enhances the specificity of the reaction, as an additional match with the target DNA will be required for a positive signal. There is, however, still a risk for false positive results due to cross-reactions and positive results need to be confirmed.

To confirm PCR positives for MAP, one of the most extensively used methods is sequencing of a part of IS900 [25-27]. However, with current generally available technology, sequencing may not be the most practical method. To obtain a successful sequencing reaction, a relatively large amount of pure amplicon is needed, which weakly infected samples might not yield. Furthermore, sequencing is a laborious and expensive method [28], not suitable for confirmation of numerous positive samples. The sensitivity of real-time PCR makes it easy to use the original template DNA in reactions targeting other sites on IS900 or on other genes. Only when the template contains minute traces of MAP DNA or is slightly PCR inhibiting, can problems arise, as discussed below.

The two systems DH1 and DH2 were selected on the basis of minimal sequential similarity to related IS elements in other mycobacteria, such as the IS900 equivalent in strain 2333 [20], and minimal self-complementarity. It appears that the sequential similarity to strain 2333 predominates among the first 450 base pairs of IS900, which is also the area in which most of the systems suggested by the Primer3 software are found. Most previously described probe-based real-time PCR systems on the IS900 gene are also located there and thus, they have little or no possibility to discriminate against 2333 [29-32]. Many potential systems could therefore be excluded, after which DH1 and DH2 were selected from the remaining oligos. In contrast to IS900, F57 has no known similarities to genes on other related organisms, which made the task of selecting suitable oligos for F57 less complicated.

It may appear simpler to change the primary system to one of the new systems, as they seem to be more specific than the MP system, but it should be noted that it is not certain that each of the other systems alone is entirely specific either. When using only one of the other systems, there is instead a risk of cross-reactions with other, unknown organisms with similarities in other parts of the MAP genome. For example, only a limited number of mycobacteria that do not grow at 37°C have been tested with F57 systems. The four systems eliminate many potential false positives by complementing each other, covering different parts of the IS900 – or, as for the DH3, a part of the F57 gene. However, the DH3 together with the primary system would probably be sufficiently specific for the routine application, where DH1 and DH2 only need to be used if DH3 fails, as discussed below.

Previously published works on systems targeting F57 [33-35] have reported specificity for MAP and application of their systems on altogether 95 strains of MAP and 188 other strains. Our system DH3, targeting F57, did not produce any false positives when applied to 112 strains of MAP and 155 other strains. It is however less sensitive than the systems on IS900, as there are 15 to 20 copies of IS900 in the MAP genome [3] – and specifically 17 copies in strain K-10 [36]. This is consistent with the results of our sensitivity tests. A suspension of 0.1 MAP genomes/μl contains about 1.7 IS900 elements/μl. Since real-time PCR has been reported capable of detecting a single copy of the target gene [37-39], the successful detection of MAP in 2 to 2.5 μl of this suspension was expected. As there is only one single copy of F57 in the MAP genome, the template volume had to contain at least 1 genome for successful detection. In fact, at such low concentrations, the probability of detection drops well below 100% if an insufficient volume is tested. Use of Poisson distribution shows a 63% theoretical probability of at least 1 genome observed in 1 μl at a concentration of 1 genome/μl. In 2.5 μl of template, the probability of the same is 92% and when duplicates are run, it increases to 99.3%. Similarly, one can show that the probability of finding at least one IS900 in duplicates of 2 μl of the above-mentioned suspension (0.1 genomes/μl) is 99.9%. In reality however, any detection system is less than optimal and an occasional target copy may be lost in the process, which is why one must expect lower probability of detection at these low concentrations. DNA extracted from clinical samples may be highly complex and impure and have a slightly inhibitory effect on the PCR reaction in weak samples, even when a positive internal control indicates that inhibition should not be a problem.

In theory, the confirmatory systems could be combined and optimized to work as one single multiplex confirmatory PCR. Nevertheless, the systems were kept separate, as competition of reagents can occur in a multiplex system, thus lowering its sensitivity [40,41].

If a clinical sample proves positive with the primary system, yet any of the three confirmatory systems shows negative, an investigation into the cause of this divergence must be undertaken. If the primary system elicited a strong signal and all laboratory errors can be excluded, the likelihood is that the result was false positive, produced by some other mycobacterial strain. In that case, it would probably be of interest to further investigate this strain! The other possibility is of course that the negative system is not sensitive to all MAP strains, in spite of our extensive testing.

However, when the positive signal is very weak, there may be several reasons why some of the confirmatory systems show negative. Because the genomes are sparse and attenuated in weak samples, a positive signal may be the outcome of low probability, thus impairing reproducibility. The confirmatory systems must then be employed again, this time using more replicates. In particular, the DH3 system on F57 will have a very low probability of detection at such concentrations. In rare cases, when even repeated analyses with additional replicates yield negative results, one will have to do without confirmation with F57 and instead take other aspects into account when judging the sample as true or false positive. In fact, it was notable that the F57 system could confirm the weakest detected level of spiked faeces (10^4 ^organisms/g) at the first attempt. This was probably because the weaker concentration (10^3 ^organisms/g) was very close to the detection level of the primary system. A few weak signals were yielded by the standard PCR, but with unacceptable high CT values. Another considered explanation for occasional failure of any of the systems DH1-3 to confirm the primary system was that they might be more sensitive to inhibition than was the primary system, which contains the internal control plasmid, especially as the confirmatory systems were developed with the use of a template volume of 2.5 μl instead of 2 μl. However, no such difference in robustness has yet been shown by the authors, and it is therefore assumed that they are equally sensitive to inhibition. Ultimately, when trying to confirm a positive test, one must be certain that a weak signal in one single system is not due to laboratory contamination.

The false reactions given by the *Mycobacterium avium *strain originating from horse and the *Mycobacterium kubicae*-related isolate from cat are most likely not a problem, as they were much weaker than would be expected when using template from a resuspended colony. A high CT value (>30 cycles) by confirmation of a normal-sized colony should alert the investigator that a cross-reaction or a contamination might have occurred. One could even argue that the cut-off CT value for PCR on colonies should be lower than for direct PCR on clinical samples, which would have made the above false positives negative. It was, however, not lowered, since that would also increase the risk of occasional dismissal of colonies that are very small or partly blended with contamination flora. In contrast, when higher CT values are expected, as when direct PCR is performed on clinical samples, the above strains are unlikely to yield positive signals. But if they still do, the remaining systems will show them to be false.

## Conclusion

After validation on several mycobacterial strains and on faecal samples, our new confirmatory systems were found to be both sensitive and reliable. We recommend using the DH3 PCR on the F57 gene as the primary confirmatory test for PCR positives, but if it fails due to its lower sensitivity, then the DH1 and DH2 PCR systems can be used.

## Methods

### Laboratory strains, growth conditions and extraction of genomic DNA

MAP strains were cultured for 8 weeks on modified Löwenstein-Jensen medium with mycobactin (4 mg/l, Allied Monitor, Fayette, MO, USA). Other mycobacterial strains were cultured on Löwenstein-Jensen medium at 37°C for up to 6 weeks, except the strains *Mycobacterium marinum *(CIP 104528) and *Mycobacterium cookii *(ATCC 49103), that grow at 30° but not at 37°C.

Laboratory strains and their various origins are shown in Tables 1 and 2. The following MAP strains have defined RFLP subtypes: strain Telford 9.2 (RFLP type *S1*) from R.J. Whittington and strains 5001-1425 (RFLP type *B-C12*), P1850/1/97 (*A-C10*), 17 (*Z-C18*), M211 (*H-C1*), 5TSD (*B-C17*), 93/433 (*B-C19*), P1611-15 (*B-C13*), 4064 (*B-C1*), 1038 (*B-C12*), 6/922 (*B-C2*), 6256 (*D-C12*), 7954 (*B-C16*) 25071 (*B-C13*), 9602 (*E-C1*), 9944 (*E-C1*), K126 (*B-C17*), 6042 (*D-C12*), M212 (*B-C2*), from I. Pavlik. Isolates of MAP (*n *= 20) from Sweden have been RFLP typed as *B-C1*.

Purified MAP-DNA was obtained from ATCC 19698 (American Type Culture Collection, Rockville, MD, USA) by beadbeating with zirconia/silica beads (0.1 mm, BioSpec Products, Inc., Bartlesville, OK, USA) and phenol/chloroform extraction. However, less pure DNA for confirmation of identity with real-time PCR was obtained by centrifugation of resuspended MAP colonies, heat-killed at 99°C for 10 min.

### Field isolates

Single colonies, suspected to be MAP, were isolated from faecal cultures on modified Löwenstein-Jensen medium [42] or on Herrolds Egg Yolk medium, both supplemented with mycobactin (Table 1). Other mycobacterial isolates, obtained from various sources during veterinary routine diagnostic work at the Mycobacteria Laboratory at SVA, had been isolated on Löwenstein-Jensen, Stonebrink, or Middlebrook medium (Table 2). The geographical origin as well as animal host species are also given in Table 2 and its footnotes. Two strains (*Mycobacterium marinum *from guppie and one *Mycobacterium *species from turtle) did not grow at 37°C, but only at lower temperatures.

### Direct PCR on faeces

Template DNA for direct testing with real-time PCR was extracted from bovine faeces. The protocol involved removal of solid material from 1.0 to 1.2 g faeces, lysing of the bacteria by incubation in lysisbuffer with 20 μg proteinase K and beadbeating with zirconia/silica beads (0.1 mm, BioSpec Products, Inc.). Purification of the lysate was performed with a modified QIAamp protocol (QIAamp DNA Stool Mini Kit, Qiagen). For further details, refer to previously published work [43]. Clinical samples (107 in total) were obtained from the Czech Republic, Denmark, Sweden and from the USA. The latter (52 samples) were External Quality Assessment samples (Johne's Fecal Check Test) from National Veterinary Services, Ames, IA. Spiked faeces was also tested. It was spiked with different dilutions of dispersed MAP bacteria, washed free of excessive free DNA and carefully quantified in a microscope as previously described [16] to avoid overestimation of the analytical sensitivity. The final concentrations of MAP in the spiked faeces were 10^2 ^– 10^7 ^organisms/g.

### Real-time PCR based on IS900 and F57

Our primary real-time PCR system used to detect MAP in the samples was the previously described MP system [16], which includes an internal control for indication of PCR inhibition. When positive, the presence of MAP was confirmed with three newly constructed systems: two based on IS900 and one based on the F57 gene. The free on-line primer design software Primer3 [44] was used to find potential primers and probes. From these, the two systems DH1 and DH2 were selected on IS900 and DH3 on F57. Candidate sequences were compared with other known genes using the BLAST Sequence Analysis Tool to check for incidental similarities. Selected oligos are listed in Table 4.

The PCR mixture comprised 6.625 μl H_2_O (Sigma-Aldrich) in the case of the primary MP system and 8.625 μl H_2_O in the confirmatory systems, 2.0 μl glycerol (Sigma-Aldrich), 2.5 μl 10× PCR-buffer II (Applied Biosystems, Foster City, CA, USA), 5.0 μl MgCl_2 _(25 mmol/l, Applied Biosystems), 2.0 μl GeneAmp^® ^dNTP with UTP (2.5 mmol/l dA, C, GTP, 5 mmol/l dUTP, Applied Biosystems), 0.75 μl forward primer (10 pmol/μl), 0.75 μl reverse primer (10 pmol/μl), 0.5 μl MAP-specific probe (10 pmol/μl), 0.5 μl mimic-specific probe (10 pmol/μl) in the case of the primary MP-system, 0.125 μl AmpliTaq Gold^® ^(5 U/μl, Applied Biosystems) and 0.25 μl AmpErase^® ^(Uracil N-glycosylase, 1 U/μl, Applied Biosystems). The addition of glycerol allowed freezing of the PCR mixture. In the case of the MP system, 2 μl of template DNA and 2 μl of mimic molecule pWIC9 (150 fg/μl) [16] were added to each reaction tube, except for the PCR-negative controls. In the case of the confirmatory systems, no mimic molecule but 2.5 μl of template DNA was added.

The real-time PCR reaction was performed on a Rotor-Gene 3000 (Corbett Research, Mortlake, Australia) with the following program: 50°C 2 min, 95°C 10 min, repeat 95°C 15 s and 60°C 1 min, 45 times. The results were analysed with the Rotor-Gene software versions 5 and 6 and the built-in analytical tools Dynamic Tube Normalisation and Slope Correction. Real-time PCR curves of normalized fluorescence for FAM crossing a threshold value of 0.01 at less than 40 cycles were considered positive, as long as the curves had a normal and expected shape. FAM-negative curves with a positive corresponding ROX curve (i.e. a positive mimic signal) were considered as true negatives; otherwise, inhibition was suspected. DNA extracted directly from faeces was run in duplicates.

If a slightly infected clinical sample yielded positive results with all systems except the DH3 on F57, additional runs were performed; first with five replicates, then if still negative, with 15 replicates.

### Sensitivity test on pure DNA

The concentration of purified MAP-DNA was determined with NanoDrop (Wilmington, DE, USA) and low, specific concentrations were obtained by serial dilution. At the lowest concentrations (less than 100 genomes per μl) the DNA was diluted in half-multiples of ten (10^0.5^) for better resolution of the sensitivity measurement. Real-time PCR was run in duplicates on DNA suspension ranging from 10^-1.5 ^(~0.03) to 10^3 ^MAP genomes per μl with all four PCR systems described in Table 4.

## Authors' contributions

DH participated in the design of the study, designed the PCR systems, carried out the laboratory work and drafted the manuscript. GB conceived of the study, participated in its design and coordination and helped to draft the manuscript. All authors read and approved the final manuscript.

## Figures and Tables

**Table 1:** Strains of *Mycobacterium avium *subsp. *paratuberculosis*, tested positive with the primary and confirmatory PCR systems.

**No. of strains**	**Source**	**Origin**
77	Cattle	Various^a^
19	Goat	Various^b^
4	Sheep	Various^c^
1	Deer	Czech Rep.
1	Human	ATCC 49164
1	Human	ATCC 43015
1	Cattle	NCTC 8578
1	Human	Czech Rep.
1	Cattle	CIP 107488
1	Cattle	ATCC 19698^T^
5	Unknown	Various^d^

a. Sweden (13), USA (28), Denmark (12), Czech Rep. (16), Switzerland (1), Norway (2), Slovak Rep. (2), Argentina (2), Great Britain (1).

b. Norway(16), UK (1), Greece (1), New Zeeland (1).

c. Australia (1), USA (1), Faroe Islands (1), Czech Rep. (1).

d. Denmark (4), Czech Rep.(1).

T. Type strain

**Table 2:** Non-target strains, tested with the primary and confirmatory PCR systems.

**No. of strains**	**Species**	**Source**	**Origin**	**IS900 MP**	**IS900 DH1**	**IS900 DH2**	**F57 DH3**
1	MAA^a^	Horse	Sweden	-	**+**	-	-
1	MAA	Human	ATCC 35718	-	-	-	-
1	MAA	Domestic fowl	ATCC 25291^T^	-	-	-	-
6	MAA	Various^b^	Various^c^	-	-	-	-
1	*Mycobacterium *species (strain 2333)	Cattle	Sweden	**+**	-	-	-
1	*Mycobacterium *species^d^	Cat	Sweden	-	-	**+**	-
1	*Mycobacterium kansasii*	Human	ATCC 12478^T^	-	-	-	-
1	*Mycobacterium smegmatis*	Cattle	Great Britain	-	-	-	-
1	*Mycobacterium chelonei*	Cattle	Sweden	-	-	-	-
1	*Mycobacterium fortuitum*	Cattle	Sweden	-	-	-	-
1	*Mycobacterium bovis*	Deer	Sweden	-	-	-	-
1	*Mycobacterium bovis*	Cattle	ATCC 19210^T^	-	-	-	-
1	*Mycobacterium silvaticum*	Dove	Sweden	-	-	-	-
1	*Mycobacterium intracellulare*	Sparrow	ATCC 35767	-	-	-	-
1	*Mycobacterium intracellulare*		ATCC 13950^T^	-	-	-	-
1	*Mycobacterium intracellulare*	Cattle	ATCC 35771	-	-	-	-
1	*Mycobacterium scrofulaceum*	Human	ATCC 19981^T^	-	-	-	-
1	*Mycobacterium gordonae*	Human	ATCC 14470^T^	-	-	-	-
1	*Mycobacterium marinum*	Guppy	Sweden	-	-	-	-
2	*Mycobacterium marinum*	Dolphin	Sweden	-	-	-	-
1	*Mycobacterium malmoense*	Human	CIP 105775^T^	-	-	-	-
1	*Mycobacterium celatum*	Human	CIP 106109	-	-	-	-
1	*Mycobacterium flavescens*	Guinea pig	CIP 104533	-	-	-	-
1	*Mycobacterium komossense*	Sphagnum moss	CIP 105293^T^	-	-	-	-
1	*Mycobacterium marinum*	Saltwater fish	CIP 104528^T^	-	-	-	-
1	*Mycobacterium terrae*	Human	CIP 104321^T^	-	-	-	-
1	*Mycobacterium vaccae*	Cattle	CIP 105934^T^	-	-	-	-
1	*Mycobacterium xenopi*	Toad	CIP 104035^T^	-	-	-	-
1	*Mycobacterium *species^e^	Turtle	Sweden	-	-	-	-
1	*Mycobacterium porcinum*	Swine	CIP 105392	-	-	-	-
1	*Mycobacterium porcinum*	Bulk milk	Italy	-	-	-	-
1	*Mycobacterium hiberniae*	Soil	CIP 104537	-	-	-	-
1	*Mycobacterium cookii*	Sphagnum moss	ATCC 49103	-	-	-	-
110	*Mycobacterium *species	Various^f^	Various^g^	-	-	-	-
1	*Staphylococcus aureus*		ATCC 25923	-	-	-	-
1	*Streptococcus dysgalactiae*	Cattle	CCUG 27436	-	-	-	-
1	*Streptococcus uberis*	Cattle	CCUG 27444	-	-	-	-
1	*Escherichia coli*		ATCC 25922	-	-	-	-
1	*Arcanobacter pyogenes*	Cattle	CCUG 39326	-	-	-	-
1	*Pseudomonas aeruginosa*	Human	CCUG 17619	-	-	-	-

a. *Mycobacterium avium *subspecies *avium*.

b. Cat (1), Cattle (1), Deer (1), Sheep (3).

c. Sweden (3), Faroe Islands (1), Iceland (2).

d. *Mycobacterium kubicae*-related strain.

e. *Mycobacterium nonchromogenicum*-related strain.

f. Cattle (59), Sheep (23), Monkey (1), Deer (2), Buffalo (1), Horse (1), Elephant (3), Ciclide (1), Antelope (1), Alpaca (2), Environmental (1), Cell culture (1), Peat (1).

g. Sweden (82), Czech Republic (3), USA (11), Australia (2), Denmark (11), Great Britain (1)

T. Type strain

**Table 3:** Confirmation of clinical samples of bovine faeces, positive for MAP with the primary PCR.

**Samples**	***n***	Confirmed*	**Additional 5 replicates**†	**Additional 20 replicates**‡	**F57 failure§**
Culture-confirmed	47	41	4	2	0
**Not **culture-confirmed	9	3	5	0	1

* Confirmed by all three alternative systems, using two PCR replicates.

† Confirmed by DH3 on F57, only after running 5 additional PCR replicates.

‡ Confirmed by DH3 on F57, only after running 20 additional PCR replicates.

§Confirmation with the DH3 system failed after a total of 22 PCR replicates

**Table 4:** Primers and probes in the primary and confirmatory real-time PCR systems.

Oligo	Nucleotide sequence	5' annealing position*	Product length
MPF	5'-CCG CTA ATT GAG AGA TGC GAT T-3'	139	115
MPR	5'-CCA GAC AGG TTG TGC CAC AA-3'	253	
MP probe	5'-6-FAM-ACC TCC GTA ACC GTC ATT GTC CAG ATC A-BHQ-1-3'	231	
DH1F	5'-AAG ACC GAC GCC AAA GAC-3'	536	69
DH1R	5'-CAG AGG CTG CAA GTC GTG-3'	604	
DH1 probe	5'-6-FAM-GAT CAT CGC CGA TCA GGC CC-BHQ-1-3'	559	
DH2F	5'-GCC TTC GAC TAC AAC AAG AGC-3'	737	65
DH2R	5'-GCG TCG GGA GTT TGG TAG-3'	801	
DH2 probe	5'-6-FAM-GCC GCG CTG ATC CTG CTT ACT-BHQ-1-3'	761	
DH3F	5'-AAC TAA GCG GAT CGA CAA TTC-3'	493	80
DH3R	5'-TGG TGT ACC GAA TGT TGT TG-3'	572	
DH3 probe	5'-6-FAM-TGC AAC TCG AAC ACA CCT GGG A-BHQ-1-3'	519	

* According to deposited sequences X16293 and X70277 in GenBank
